# Resveratrol-induced antinociception is involved in calcium channels and calcium/caffeine-sensitive pools

**DOI:** 10.18632/oncotarget.14090

**Published:** 2016-12-22

**Authors:** Xiaoyu Pan, Jiechun Chen, Weijie Wang, Ling Chen, Lin Wang, Quan Ma, Jianbo Zhang, Lichao Chen, Gang Wang, Meixi Zhang, Hao Wu, Ruochuan Cheng

**Affiliations:** ^1^ Department of Thyroid Surgery, The First Affiliated Hospital of Kunming Medical University, Kunming, Yunnan Province, China; ^2^ Department of Neurology, Lianyungang Second People's Hospital, Lianyungang, Jiangsu Province, China; ^3^ Department of Neurosurgery, Huai’an First People's Hospital, Nanjing Medical University, Huai’an, Jiangsu Province, China; ^4^ Department of Clinical Pharmacy, Hangzhou First People's Hospital, Nanjing Medical University, Hangzhou, Zhejiang Province, China; ^5^ Department of Pharmaceutical Sciences, School of Pharmacy and Pharmaceutical Sciences, State University of New York at Buffalo, Buffalo, NY, USA; ^6^ Whitehead Institute for Biomedical Research, Cambridge, MA 02142, USA; ^7^ Pingyang Hospital of Traditional Chinese Medicine, Pingyang, Zhejiang Province, China; ^8^ Department of Neurology, Wenzhou People's Hospital, Wenzhou, Zhejiang Province, China

**Keywords:** resveratrol, calcium channels, antinociception, CaMKII, BDNF

## Abstract

Resveratrol has been widely investigated for its potential health properties, although little is known about its mechanism *in vivo*. Previous studies have indicated that resveratrol produces antinociceptive effects in mice. Calcium channels and calcium/caffeine-sensitive pools are reported to be associated with analgesic effect. The present study was to explore the involvement of Ca^2+^ channel and calcium/caffeine-sensitive pools in the antinociceptive response of resveratrol. Tail-flick test was used to assess antinociception in mice treated with resveratrol or the combinations of resveratrol with MK 801, nimodipine, CaCl_2_, ryanodine and ethylene glycol tetraacetic acid (EGTA), respectively. The Ca^2+^/calmodulin-dependent protein kinase II (CaMKII) and brain-derived neurotrophic factor (BDNF) levels in the spinal cord were also investigated when treated with the above drugs. The results showed that resveratrol increased the tail flick latency in the tail-flick test, in dose-dependent manner. N-methyl-D-aspartate (NMDA) glutamate receptor antagonist MK 801 potentiated the antinociceptive effects of sub-threshold dose of resveratrol at 10 mg/kg. Ca^2+^ channel blocker, however, abolished the antinociceptive effects of resveratrol. In contrast to these results, EGTA or ryanodine treatment (i.c.v.) potentiated resveratrol-induced antinociception. There was a significant decrease in p-CaMKII and an increase in BDNF expression in the spinal cord when combined with MK 801, nimodipine, ryanodine and EGTA. While an increase in p-CaMKII level and a decrease in BDNF expression were observed when high dose of resveratrol combined with CaCl_2_. These findings suggest that resveratrol exhibits the antinociceptive effects by inhibition of calcium channels and calcium/caffeine-sensitive pools.

## INTRODUCTION

Resveratrol (3, 4′, 5-trihydroxystilbene), a naturally occurring polyphenol compound naturally present in significant amounts in several plants, has garnered considerable interest given its presence in berries, peanuts, grapes, and red wine [1]. There are two isomers of resveratrol, *cis*-(Z) and *trans*-(E) resveratrol. *cis*-resveratrol was considered biologically inactive; *trans*-resveratrol seems to be more potent than *cis*-resveratrol in most of the comparative studies indicating certain stereo-specific activity of the molecule. Previous study also reports that both isoforms possess biological activity [2] and inhibit synthesis of proinflammatory mediators by suppressing cyclooxygenase and lipoxygenase pathways, which suggest that resveratrol may have analgesic activity [3–5].

Calcium (Ca^2+^) channel is a second messenger, which mediates a variety of intracellular functions including gene expression, neurotransmitter release and protein phosphorylation. It has been reported that calcium ion has a physiological role in the inhibition of calcium movement, which contributes to antinociception [6–11]. L-type Ca^2+^ channel antagonists, nimodipine for example, produces analgesic effect after central and peripheral administration [12–15]. Moreover, the effects of L-type Ca^2+^ channel inhibitors on nociception differ depending on the drug, dosage, and route of administration and the algesimeter test used [16]. Intracellular Ca^2+^ pools contained in the endoplasmic reticulum play a major role in tightly regulating the levels of intracellular free Ca^2+^ in cells. The Ca^2+^/calmodulin-dependent protein kinase II (CaMKII) and brain derived neurotrophic factor (BDNF) are involved in the oxaliplatin-induced mechanical allodynia [17], and also plays an important role in brain cells when talked about pain [18].

The present study attempted to find the analgesic effect of resveratrol through Ca^2+^ ion channels, especially the L-type calcium channels and calcium/caffeine sensitive pool and the regulation of pain associated p-CaMKII and BDNF. We found that calcium ions play in an important role in the analgesic effects of resveratrol, while reducing the intracellular calcium concentration, resulting in significant analgesic effect; on the contrary, the analgesic effect weakened.

## RESULTS

### Resveratrol produced antinociceptive effects in the tail flick test

**Figure 1:**
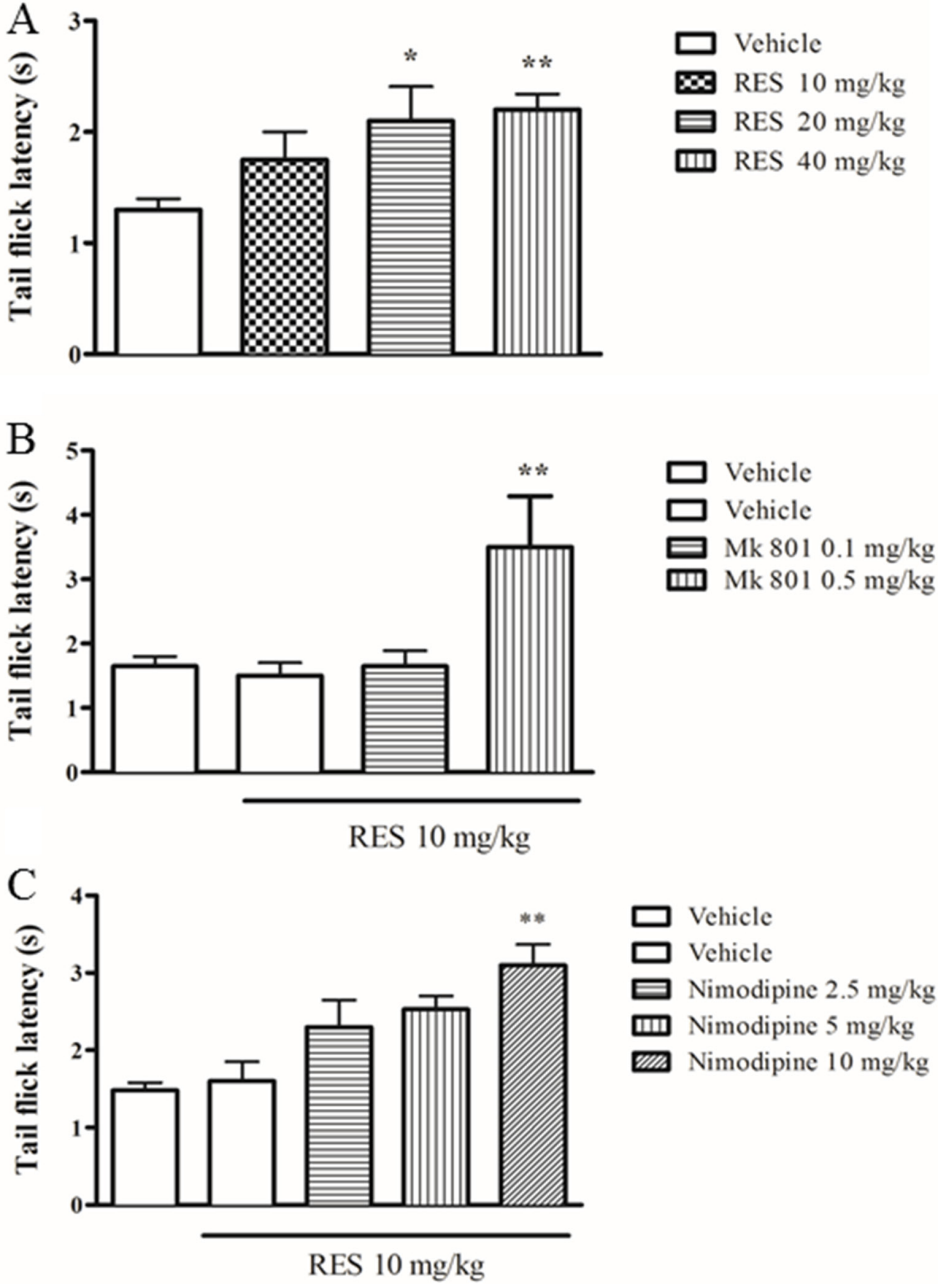
The effect of resveratrol on tail flick latency in mice Mice were administered vehicle, resveratrol (10, 20, 40 mg/kg, p.o.) 1 h before testing. Mk 801 (0.1 and 0.5 mg/kg, i.p.) and nimodipine (2.5, 5 and 10 mg/kg, i.p.) were administered 30 min before resveratrol treatment. Mean ± SEM, *n* = 8. *p < 0.05 and **p < 0.01, compared to the vehicle+resveratrol (10 mg/kg) group.

To confirm the analgesic effect of resveratrol and whether NMDA receptor antagonist MK 801 is involved in the effect of resveratrol, we examined antinociceptive response of resveratrol in the tail-flick test in mice treated with resveratrol or combined with MK 801. Resveratrol at doses of 10∼40 mg/kg increased the latency to a powerful light beam in the tail-flick test, in a dose-dependent manner (F = 3.26, *p* < 0.01) (Figure 1A). MK 801, an N-methyl-D-aspartate (NMDA) receptor inhibitor, at the dose of 0.5 mg/kg, increased the latency to nociceptive stimulus when combined with resveratrol although it did not induce any behavioral changes when it was used alone (*p* < 0.01) (Figure 1B), which were consistent with the previous studies [19, 20].

To further determine whether L-type Ca^2+^ channels are involved in the antinociception induced by resveratrol, the effect of nimodipine on the inhibition of nociceptive response was examined. Nimodipine at 2.5, 5 and 10 mg/kg (i.p.) treated alone did not show any effects in the tail flick latency in mice compared to the vehicle-treated group (data now shown), which was in agreement with the previous findings [21]. However, pretreatment with nimodipine potentiated the effects of resveratrol under sub-threshold dose at 10 mg/kg (F = 6.49, *p* < 0.05) (p.o.) (Figure 1C).

### CaCl_2_ reversed resveratrol's effect, while EGTA and ryanodine potentiated the antinociceptive effects of resveratrol

**Figure 2:**
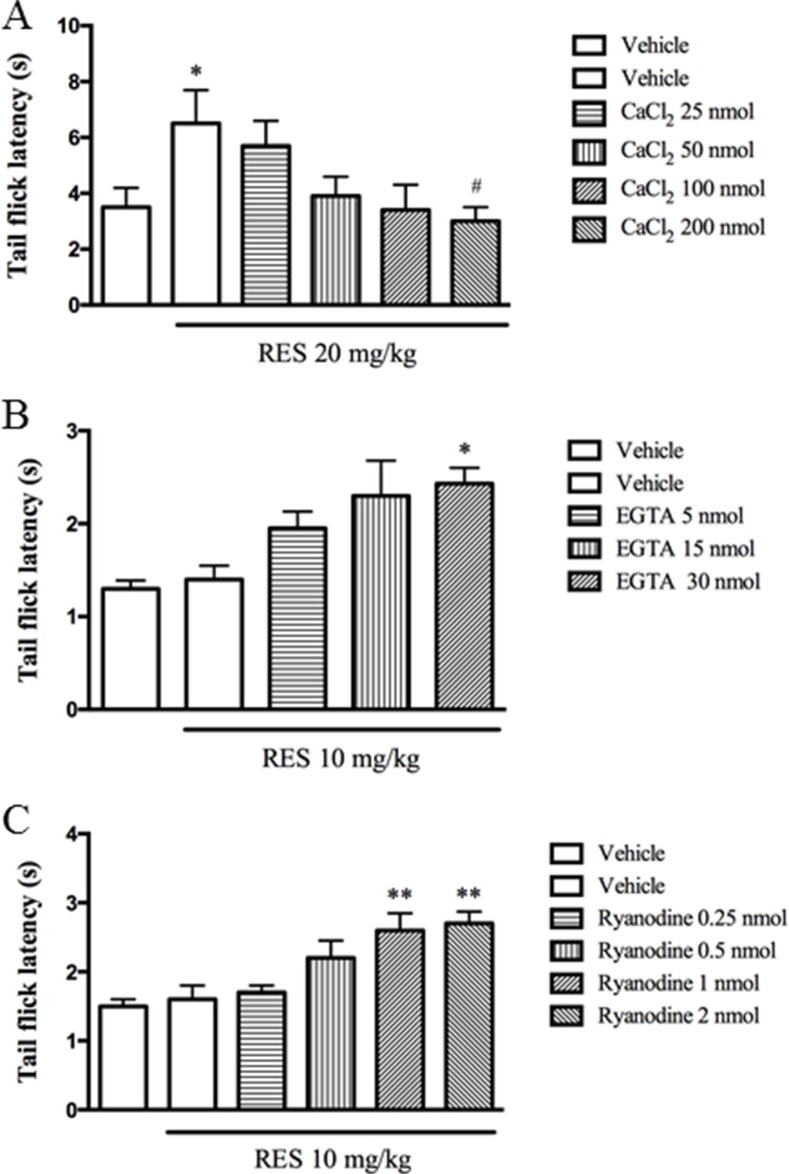
CaCl2 reversed resveratrol's effect, while EGTA and ryanodine potentiated the antinociceptive effects of resveratrol CaCl_2_ (25, 50, 100 and 200 nmol, i.c.v), EGTA (5, 15 and 30 nmol, i.c.v) and ryanodine (0.25, 0.5, 1 and 2 nmol, i.c.v) were administered 30 min before resveratrol treatment. Mean ± SEM, *n* = 8. *p < 0.05 and **p < 0.01, compared to the vehicle + resveratrol (10 or 20 mg/kg) group.

The subsequent study explored how the i.c.v. administration of Ca^2+^ or EGTA (a selective Ca^2+^ chelator) affects the antinociceptive effects induced by resveratrol. CaCl_2_ (25, 50, 100 and 200 nmol) alone did not induce the antinociception or other behavioral abnormality in the preliminary study. However, i.c.v. administration of CaCl_2_ at doses of 25∼200 nmol significantly reversed the antinocicetive responses induced by resveratrol at 20 mg/kg (F = 3.21, *p* < 0.05) (Figure 2A). By contrast, i.c.v. injection of EGTA (5, 15 and 30 nmol) increased the tail flick latency when combined with the 10 mg/kg resveratrol (F = 2.80, *p* < 0.05) (Figure 2B), but it did not show any behavioral changes when it was used alone in our preliminary study.

Previous *in vitro* studies indicated ryanodine inhibits Ca^2+^ release from intracellular microsomal pools [22, 23]. The present study explored whether inhibition of Ca^2+^ release from microsomal pools by ryanodine involves resveratrol's antinociceptive effects. Ryanodine (0.25∼2 nmol, i.c.v.) used alone was not observed the antinociceptive or toxic effects in our preliminary study. However, ryanodine at doses of 0.25, 0.5, 1 and 2 nmol increased the tail flick latency in dose-dependent manner when combined with 10 mg/kg resveratrol (F = 4.91, *p* < 0.05) (Figure 2C).

### The effect of resveratrol on p-CaMKII and BDNF expression in the spinal cord

**Figure 3:**
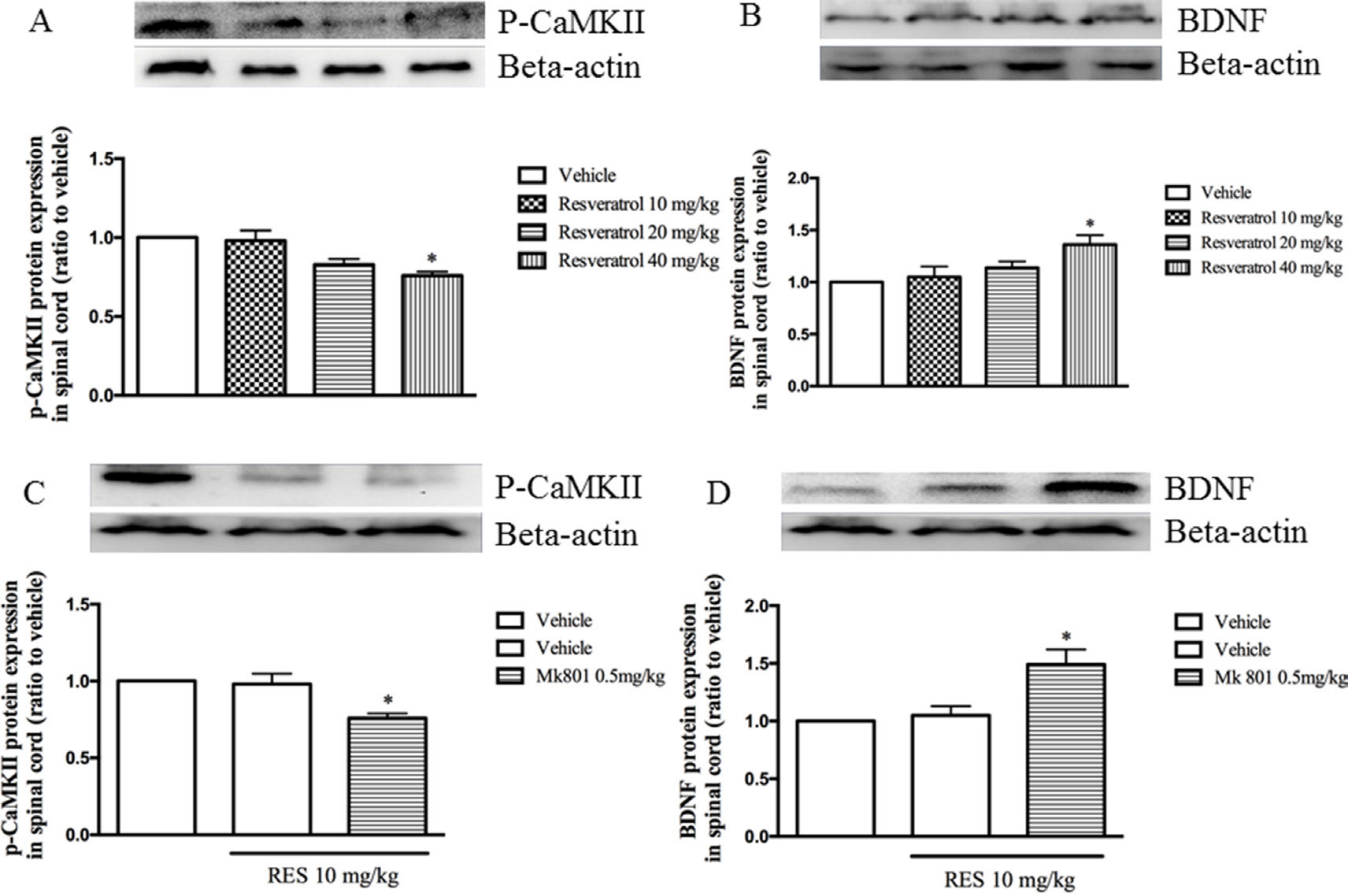
The effect of resveratrol on p-CaMKII and BDNF expression in the spinal cord (**A** and **B**) and Mk 801 potentiated resveratrol's effect on p-CaMKII and BDNF expression (**C** and **D**). Mean ± SEM, *n* = 8. *p < 0.05, compared to the vehicle + resveratrol (10 mg/kg) group.

As shown in Figure 3A and 3B, resveratrol at doses of 10, 20 and 40 mg/kg decreased p-CaMKII expression (F = 7.03, *p* < 0.05) and increased BDNF level in the spinal cord (F = 3.65, *p* < 0.05). MK 801 at doses of 0.1 and 0.5 mg/kg used alone did not show to affect both of p-CaMKII and BDNF expression (data not shown). However, the effects on p-CaMKII and BDNF expression were potentiated when combined with low dose of resveratrol at 10 mg/kg, i.e. decreased p-CaMKII and increased BDNF levels in the spinal cord were observed in the spinal cord as shown in Figure 3C and 3D (F = 7.34, *p* < 0.05; F = 6.89, *p* < 0.05).

### Nimodipine potentiated the effects of resveratrol on p-CaMKII and BDNF expression in the spinal cord

**Figure 4:**
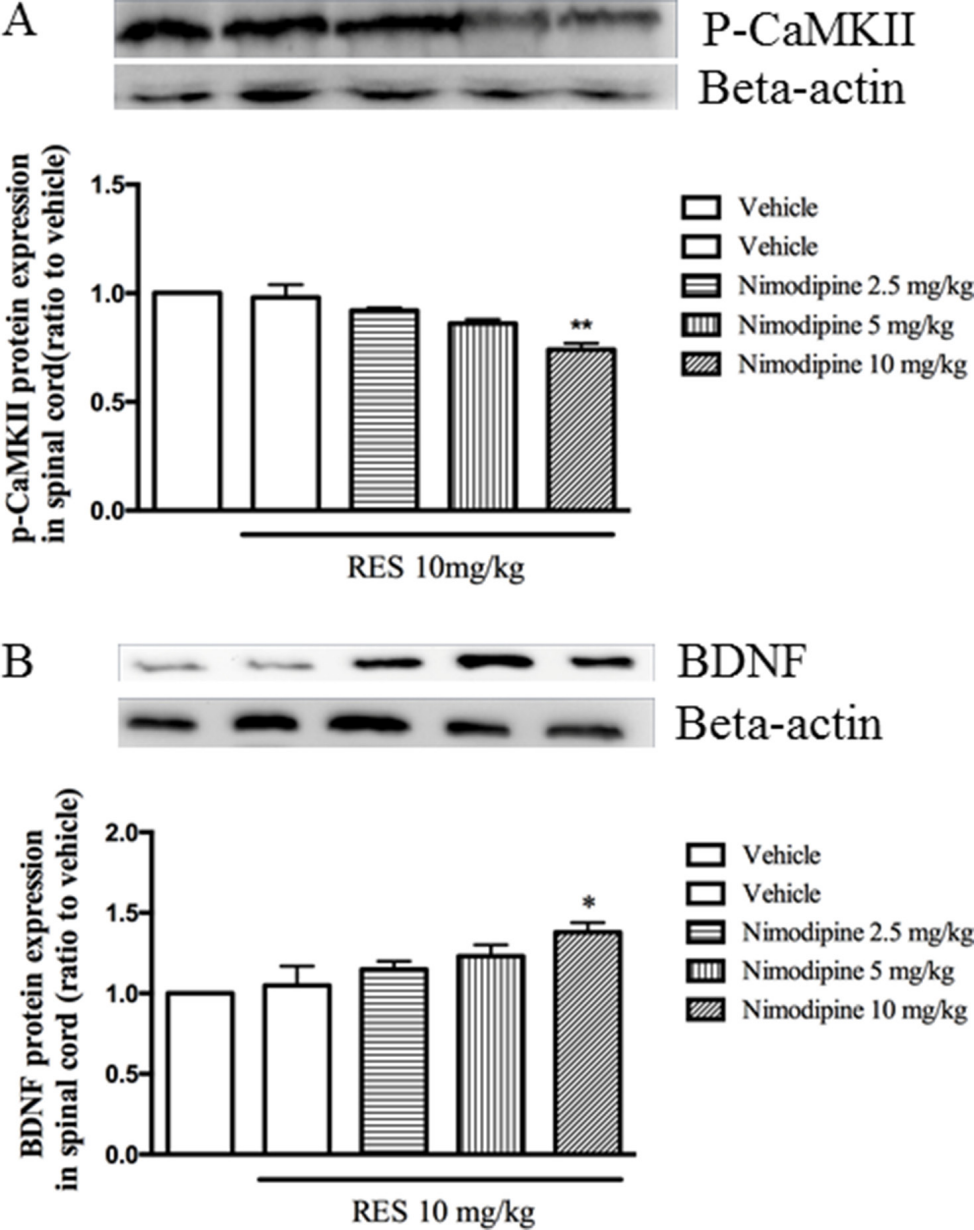
Nimodipine potentiated the effects of resveratrol on p-CaMKII and BDNF expression in the spinal cord Mean ± SEM, *n* = 8. *p < 0.05 and *p* < 0.01, compared to the vehicle+resveratrol (10 mg/kg) group.

Low dose of resveratrol at 10 mg/kg did not affect both p-CaMKII and BDNF expression as shown in Figure 3. However, pretreatment with nimodipine at doses of 2.5, 5 and 10 mg/kg potentiated resveratrol's effects on p-CaMKII and BDNF expression in the spinal cord, in a dose-dependent manner (F = 7.75, *p* < 0.05; F = 7.66, *p* < 0.05; Figure 4A and 4B). But these doses of nimodipine did show any effects on the above two proteins expression when it was used alone in our preliminary data.

### CaCl_2_ reversed, but EGTA potentiated resveratrol's effects on pCaMPKII and BDNF expression in the spinal cord

**Figure 5:**
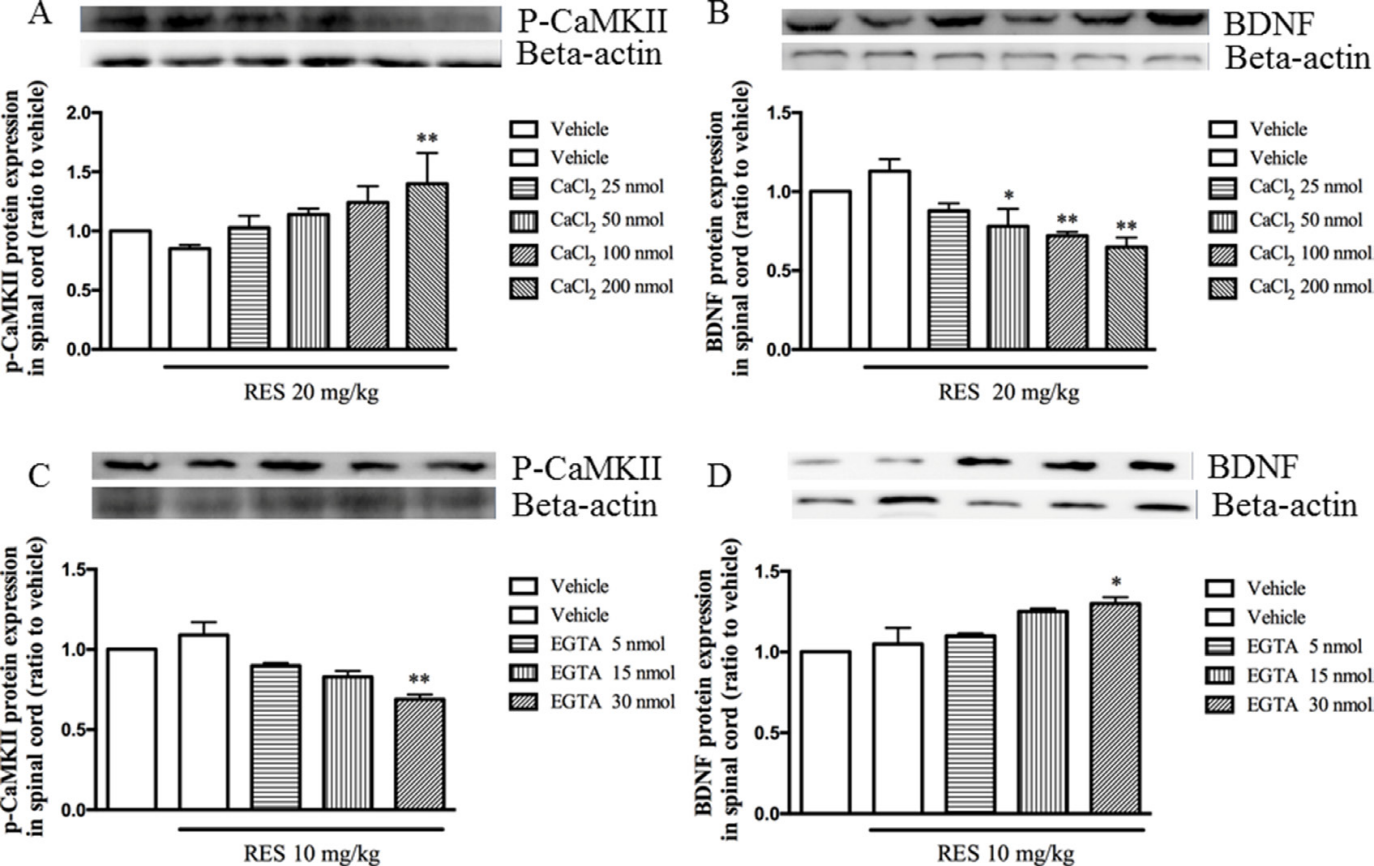
CaCl2 reversed, but EGTA potentiated resveratrol's effects on pCaMPKII and BDNF expression in the spinal cord Mean ± SEM, *n* = 8. *p < 0.05 and *p* < 0.01, compared to the vehicle + resveratrol (20 or10 mg/kg) group.

To confirm the effects of resveratrol on p-CaMKII and BDNF expression in the spinal cord are related to the calcium channel, CaCl_2_ and Ca^2+^ channel selective chelator EGTA were administered by i.c.v and the effects of them on levels of p-CaMKII and BDNF were observed. The results showed that the i.c.v. pre-injection CaCl_2_ reversed 20 mg/kg resveratrol-induced decreased p-CaMKII expression, i.e. p-CaMKII expression was increased after i.c.v. pre-treatment with CaCl_2_ (F = 3.19, *p* < 0.05) (Figure 5A). Pre-injection with CaCl_2_ 30 min before 20 mg/kg resveratrol treatment induced a dose-dependent decrease in BDNF expression in the spinal cord (F = 7.53, *p* < 0.05) (Figure 5B). However, pretreatment with different doses of selective Ca^2+^ channel chelator EGTA potentiated the effect of low dose of resveratrol (10 mg/kg) on p-CaMKII expression, which showed a significant dose-dependent decreased p-CaMKII levels in the spinal cord (F = 7.03, *p* < 0.05) (Figure 5C). Pretreatment of EGTA also potentiated low dose of resveratrol induced BDNF expression (F = 7.66, *p* < 0.05) (Figure 5D), which confirmed the extracellular calcium ions participated resveratrol's effects on noxious stimulation.

### Ryanodine potentiated the effect of resveratrol on p-CaMKII and BDNF expression

**Figure 6:**
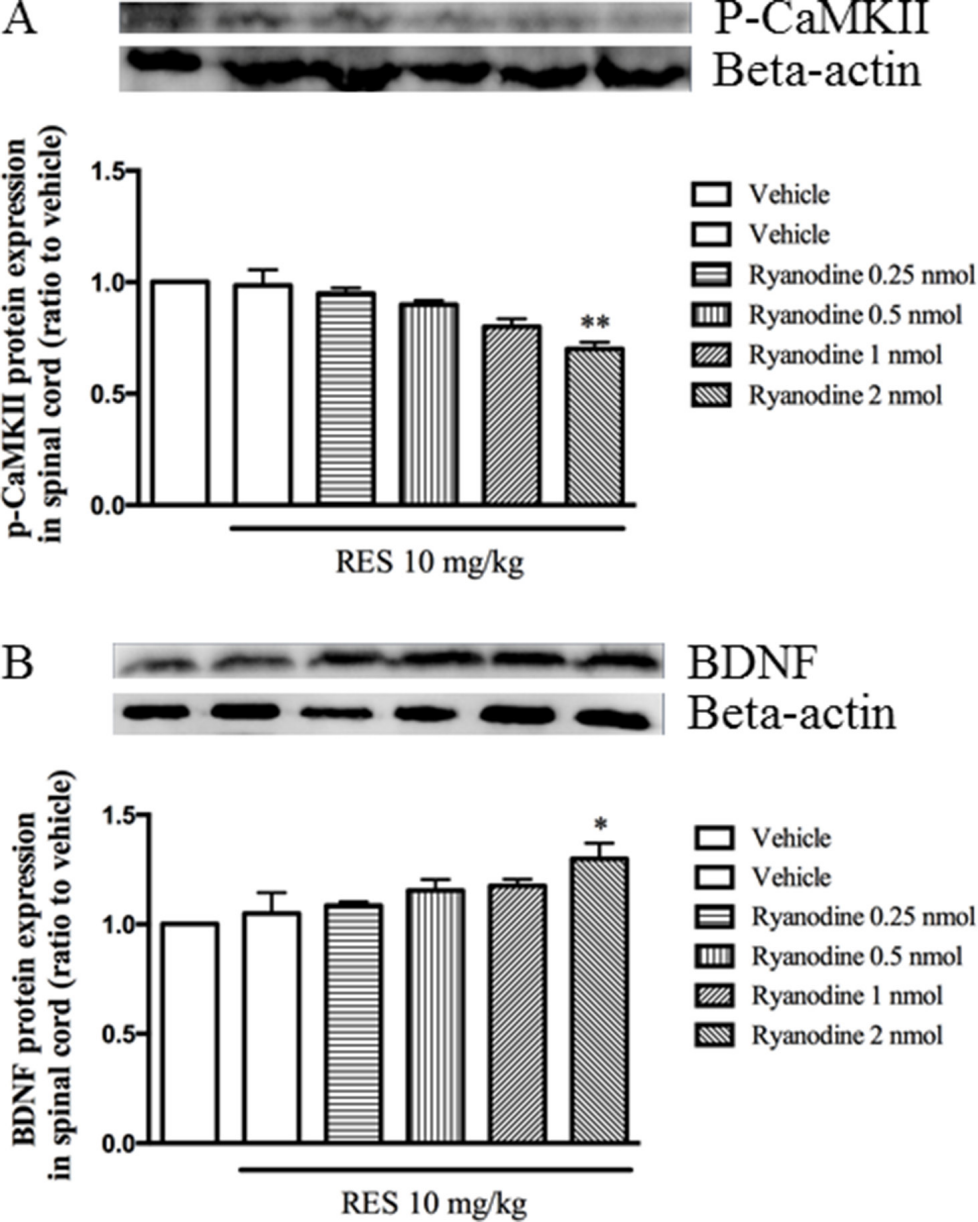
Ryrandine potentiated the effects of resveratrol on p-CaMKII and BDNF expression in the spinal cord Mean ± SEM, *n* = 8. *p < 0.05 and *p* < 0.01, compared to the vehicle+resveratrol (10 mg/kg) group.

To verify the involvement of the calcium ions released from calcium/caffeine-sensitive pools in the effects of resveratrol, the expression of p-CaMKII and BDNF in the spinal cord were detected when pretreatment with ryanodine 30 min before resveratrol was given. Figure 6A showed that ryanodine significantly enhanced low dose of resveratrol's effect on p-CaMKII, i.e. significantly decreased p-CaMKII expression when pretreatment with ryanodine (F = 6.93, *p* < 0.05). However, BDNF expression was increased significantly when low dose of resveratrol combined with ryanodine (F = 5.62, *p* < 0.05) (Figure 6B). However, different doses of ryanodine used alone did induce any effects on these two proteins expression (data not shown).

## DISCUSSION

Pain affects about 20% of the world's population, which is a great economic and social burden to community [24, 25]. Neuropathic pain is the main type of pain. Most of neuropathic pain is due to nervous system damage and dysfunction [26]. Due to the central and peripheral neuralgia complex mechanisms, therapeutic agents that can be used is limited, and these drugs often require large doses, which greatly limits their use. Currently, the use of analgesic drugs can only alleviate the 40–50% of pain for 30–40% of patients [27].

Calcium ions (Ca^2+^) enter into cell through voltage-gated calcium channels (VGCCs) excitement, and VGCCs mediated calcium channel is an initial step of presynaptic nerve terminals neurotransmitter release. Because of their special role in calcium signaling, VGCCs is an important target for the treatment of pain. Ca^2+^ as an intracellular second messenger is involved in a series of functional activities, including electrophysiological activity, neurotransmitter release, protein phosphorylation and gene expression [21]. Central Ca^2+^ is closely related to the neurotransmitter transmission of pain, such as tail-flick test. Oral administration of L-type calcium channel antagonists and nimodipine can improve the effect on pain threshold [14, 28]. Furthermore, research has shown that Ca^2+^/caffeine sensitive microsomal pool, calmodulin and CaMKIIK participate in β-endorphin and morphine analgesia [29]. Increase in the Ca^2+^ levels can antagonize the analgesic effect of opioid. Ca^2+^ and Ca^2+^ vectors such as X-537A and A23187 will shift Ca^2+^ from extracelluar to intracellular, thereby increasing the intracellular Ca^2+^ concentration, decreased analgesic effect of opioids [30, 31]. Intracerebral injection of Ca^2+^ chelator EGTA can enhance the analgesic effect of opioids [32, 33]. Ca^2+^ is very easy to penetrate from the NMDA receptor channel into intracellular [34, 35]. NMDA receptor channel has two distinct gated modes of ligands and voltage-gated. In the resting potential Mg^2+^ channel was blocked by voltage-gated [37, 38, 39]. When the membrane depolarization, NMDA receptor channel opening, calcium ions enter into intracellular. Some researchers found that nerve compression can be reversed by resveratrol in a dose-dependent manner by mechanical hypersensitivity and thermal hyperalgesia tests [36]. The present study suggested that resveratrol significantly increased the pain threshold in mice in the tail-flick test. The N-methyl-D-aspartate (NMDA) receptor inhibitor MK 801 increased the latency to nociceptive stimulus when combined with resveratrol, which are consistent with the previous studies.

Given low dose of L-type calcium channel antagonist nimodipine does not produce analgesic effects [21], but nimodipine significantly enhanced resveratrol's analgesic effects with subthreshold dose (10 mg/kg). Nimodipine antagonizes L-type calcium channel to reduce intracellular calcium ion concentration, which are in agreement with our findings that suggested that nimodipine enhanced the analgesic effect of resveratrol. The present results also suggested that intracerebral injection of CaCl_2_ reversed the analgesic effects of resveratrol, but selective calcium ion chelator EGTA enhanced the analgesic effect of resveratrol. Previous studies demonstrated that the reduced concentration of calcium ions in neurons could produce an analgesic effect, while increased intracellular calcium ion concentration could antagonize the analgesic effect [40]. Contrary to intracerebral injection of calcium ion chelator EDTA, direct injection of CaCl_2_ can increase synaptic calcium concentration, and enable neurons to have function [41]. In our present study, CaCl_2_ was injected to the cerebral ventricle, which increased extracellular calcium concentration, then increased the calcium ion permeable membranes into cells, thereby increasing the intracellular calcium ion concentration, blocking the analgesic effect of resveratrol. Similar to L-type calcium channels antagonist, intracerebral injection of calcium ion selective chelators EGTA reduced the concentration of extracellular calcium and calcium ions to enter cell membrane, leading to the reduction of intracellular calcium concentration, thereby enhancing the analgesic effect of resveratrol. Indeed, calcium concentration in neurons affects not only by calcium ions regulation that permeable membrane from extracellular into intracellular, but also by calcium ions regulation that influences of calcium stores by inositol triphosphate and ryanodine receptor in cells. Ryanodine receptor plays an important role in the regulation of calcium store and microsomal calcium release [23]. Saeki et al. [42] found that the brain-type ryanodine receptor expression in the hamster is always calcium and caffeine sensitive. In our experiments, intracerebral injection of ryanodine significantly enhanced the analgesic effects of resveratrol. Studies have shown that ryanodine blocks the release of calcium from calcium/caffeine sensitive microsomal pool [23]. Injection with ryanodine allows intracellular release of calcium ions to decrease the store of calcium, which enhances the analgesic effect of resveratrol. In addition, ryanodine also reduces the rate of calcium ions from the extracellular into the intracellular [43], which also helps to enhance its potentiation on the analgesic effect of resveratrol.

CaMKII is a serine/threonine kinase involved in a variety of neurological function. When intracellular Ca^2+^ signal markedly enhances [44], calcium ions will bind to Ca^2+^ binding sites by calmodulin after activation of CaMKII phosphorylation, which causes pain. Studies have shown that CaMKII first focuses on the area of pain transmission, such as superficial spinal dorsal horn and dorsal root ganglia [45]. Animal studies have shown that it is involved in the formation and transfer of peripheral neuropathic pain [46]. In the present study, when given resveratrol or administered simultaneously MK 801, nimodipine, EGTA and ryanodine, the expression of p-CaMKII in the spinal cord was significantly reduced; and when the intracerebral administration of CaCl_2_, the p-CaMKII expression in the spinal cord was significant increased. Previous studies suggested that excessive Ca^2+^ or CaMKII over phosphorylation might produce some degree of neurotoxicity to the cells and inhibit some biochemical pathways such as CREB and BDNF signaling [47]. When excessive Ca^2+^ flows into intracellular by L-type voltage-gated calcium channels or NMDA receptors, BDNF transcription was inhibited [48, 49, 50, 51]. Previous work suggested that peripheral nerve injury, such as the tail-flick test, is an expression of neuropathic pain, which is alleviated when the increase in BDNF release [52]. Our findings suggest that BDNF expression may directly inhibit the perceptive behaviors by the tail flick test. Pre-administration with MK 801, nimodipine, EGTA and ryanodine, BDNF expression in the spinal cord was significantly increased, while the intracerebral injection of CaCl_2_ decreased the BDNF expression. However, further studies may be needed to confirm whether the nociceptive effect of resveratrol can be inhibited by BDNF directly or indirectly.

In summary, the increase in the intracellular Ca^2+^ can potentiate resveratrol's analgesic effect significantly, while the increase in the intracellular Ca^2+^reversed resveratrol's effect on nociception. CaMKII and BDNF in the spinal cord participate the effects of resveratrol on the nociceptive response. These findings implicate that calcium channels and the calcium/caffeine-sensitive pool are involved in the analgesic effects of resveratrol.

## MATERIALS AND METHODS

### Animals

Male ICR mice (25–30 g) bred in the Animal Center of Shanghai Branch of Chinese Academy of Sciences were used. On arrival, the animals were housed under standard laboratory conditions, maintained on a 12 h natural light/dark cycle and free access to food and water. Ambient temperature and humidity were maintained at 23–25°C and 50 ± 10% respectively. Before the tests, animals were acclimatized to laboratory conditions for 1 week. The experiments were performed with 10 mice per treatment group according to a randomized schedule between 10:00 h and 18:00 h. All experiments were conducted in accordance with the National Institutes of Health Guide for Care and Use of Laboratory Animals (Publication No. 85–23, revised 1985), and approved by the Wenzhou Medical College Committee on Animal Care and Use.

### Drugs and chemicals

The drugs and chemicals used included resveratrol (Wuhan St. Tianyu Technology Co., Ltd., China), nimodipine (Enzo Biochem, Inc., USA), CaCl_2_ (Quzhou Juhua Reagent Co., Ltd., China), ethylene glycolbis (ß-aminoethyl-ether)-N, N, N’, N’-tetraacetic acid (EGTA)(Sinopharm Chemical Reagent Co., Ltd., China), ryanodine (Tocris Bioscience, UK), Mk 801 (Sigma, USA). For oral administration (via gavage, p.o. with a vloume of 10 ml/kg), transe-resveratrol was dissolved in 0.5% sodium carboxymethyl cellulose (CMC-Na) and diluted to the desired concentration on the day of testing [53]. The other drugs and chemicals were dissolved in 0.9% saline with the exception of nimodipine, which was dissolved in one drop of ethanol and diluted with saline, and EGTA, which was dissolved in sterilized H_2_O [21]. CMC-Na or saline was used as vehicle control in all the experiments. Nimodipine and Mk 801 were injected i.p. in a volume of 10 ml/kg, while CaCl_2_, EGTA and ryanodine were given by i.c.v. administration.

### Drug treatment

The i.c.v. injections were carried out as described previously [54, 55].Vehicle or drugs were injected in a volume of 1 μl into the mouse hippocampus CA1 [56]. In short, during anesthesia, mice were grasped firmly by the loose skin behind the head. A 27-gauge needle attached to a 50 μl Hamilton syringe was inserted perpendicularly through the skull into the brain, no more than 2 mm, and 1 μl of solution was injected. The injection site was 2 mm from either side of the midline drawn through the anterior base of the ears. Injections were performed into the hippocampus CA1. To verify the injection sites, a volume of 10 μl of 1% methylene blue was injected and the brains were sectioned and studied histologically.

### Behavioral tests

The tail flick test is an acute model of pain, which was used to assess the anti-nociceptive effect of the drugs by measuring the latency of response to a light beam [57]. In this method, animal tail was exposed to a powerful light beam and the response latency period for flicking the tail off the beam was recorded [58]. We applied radiant heat to the tail at 5–8 cm from the tip by using a tail flick apparatus. Tail flick latency time was estimated as the time from the onset of the heat exposure to the withdrawal of the tail. They adjusted the intensity of radiant heat to yield the baseline latencies of 2–4 second. In order to avoid tissue damages the heat stimulus was discontinued after 7 second (Cut off point = 7 sec). In this study, tail flicking test was performed 30 min after injection (20).

### Immunoblot analysis

Tissue samples from lumbar enlargement were dissected and stored at −80°C until analysis. The supernatant fluid containing 60 μg of protein was electrophoresed in a polyacrylamide gel and blotted onto PVDF membranes. The membranes were incubated with 5% non-fat dried milk in Tris buffer saline containing Tween 20 for 1.5 hours at room temperature (25°C) and then incubated with rabbit anti-CaMKII monoclonal antibody (1: 1000, Cell Signaling Technology, USA) or with rabbit anti-phosphorylated CaMKII polyclonal antibody (1: 1000, Cell Signaling Technology, USA) or with rabbit anti-BDNF polyclonal antibody (1: 1000, Abcam, USA) at 4°C overnight. Following three washing with TBST, the membrane was then incubated with an HRP-conjugated monkey anti-rabbit IgG (1:10000, Santa-Cruz) for CaMKII or p-CaMKII or BDNF for 1 hour at room temperature. Finally, membranes were washed again with Tris buffer saline containing Tween 20 to remove unbound secondary antibodies and visualized using ChemiDoc XRS System. The blots were then incubated with a polyclonal rabbit anti-β-actin antibody (1:1000, Cell Signaling Technology, USA) as the loading controls. Each experiment was repeated at least once and the same results were obtained in all cases. Six animals per group were used in the Western blotting analysis. The density of specific bands was measured using a computerized image analysis system.

### Statistical analysis

All data were presented as mean ± standard error (SEM) or and statistically analyzed with repeated measures analysis of variances (ANOVA) followed by Dunnett's post hoc tests. Differences were considered to be statistically significant at *P* < 0.05.
